# The psychophysiological effects of Tai-chi and exercise in residential Schizophrenic patients: a 3-arm randomized controlled trial

**DOI:** 10.1186/1472-6882-14-364

**Published:** 2014-09-27

**Authors:** Rainbow Tin Hung Ho, Adrian Ho Yin Wan, Friendly So Wah Au-Yeung, Phyllis Hau Yan Lo, Pantha Joey Chung Yue Siu, Cathy Pui Ki Wong, Winnie Yuen Han Ng, Irene Kit Man Cheung, Siu Man Ng, Cecilia Lai Wan Chan, Eric Yu Hai Chen

**Affiliations:** Centre on Behavioral Health, The University of Hong Kong, 2/F, The Hong Kong Jockey Club Building for Interdisciplinary Research, 5 Sassoon Road, Pokfulam Hong Kong, China; Department of Social Work and Social Administration, The University of Hong Kong, The University of Hong Kong, Room 534, Jockey Club Tower, The Centennial Campus, Hong Kong, China; The Providence Garden for Rehab, Hong Kong Sheng Kung Hui Welfare Council Limited, No. 82, Tsun Wen Road, Tuen Mun, New Territories Hong Kong; Department of Psychiatry, The University of Hong Kong, Queen Mary Hospital, 102 Pokfulam Road, Hong Kong, China

**Keywords:** Tai-chi, Exercise, Schizophrenia, Chinese, Randomized controlled trial (RCT), Salivary cortisol

## Abstract

**Background:**

Patients with schizophrenia are characterized by high prevalence rates and chronicity that often leads to long-term institutionalization. Under the traditional medical model, treatment usually emphasizes the management of psychotic symptoms through medication, even though anti-psychotic drugs are associated with severe side effects, which can diminish patients’ physical and psychological well-being. Tai-chi, a mind-body exercise rooted in Eastern health philosophy, emphasizes the motor coordination and relaxation. With these potential benefits, a randomized controlled trial (RCT) is planned to investigate the effects of Tai-chi intervention on the cognitive and motor deficits characteristic of patients with schizophrenia.

**Methods/design:**

A 3-arm RCT with waitlist control design will be used in this study. One hundred and fifty three participants will be randomized into (i) Tai-chi, (ii) exercise or (iii) waitlist control groups. Participants in both the Tai-chi and exercise groups will receive 12-weeks of specific intervention, in addition to the standard medication and care received by the waitlist control group. The exercise group will serve as a comparison, to delineate any unique benefits of Tai-chi that are independent of moderate aerobic exercise. All three groups will undergo three assessment phases: (i) at baseline, (ii) at 12 weeks (post-intervention), and (iii) at 24 weeks (maintenance). All participants will be assessed in terms of symptom management, motor coordination, memory, daily living function, and stress levels based on self-perceived responses and a physiological marker.

**Discussion:**

Based on a promising pilot study conducted prior to this RCT, subjects in the Tai-chi intervention group are expected to be protected against deterioration of motor coordination and interpersonal functioning. They are also expected to have better symptoms management and lower stress level than the other treatment groups.

**Trial registration:**

The trail has been registered in the Clinical Trials Center of the University of Hong Kong (HKCTR-1453).

## Background

### Effectiveness of exercise interventions in psychiatric disorders

The traditional medical model of schizophrenia prioritizes self-care and management of symptoms and functional abilities, both in daily patient care and in research; physical and psychological well-being are considered secondary treatment goals. Patients with schizophrenia have comparatively shorter life expectancies than the general population. This is thought to be due to physical factors (such as higher rates of cardiovascular and metabolic diseases, including obesity) as well as psychological factors (i.e. depression and suicidality) [1–4]. However, these outcomes may be partially attributable to the side effects of medication and poor lifestyle factors such as high-fat low-fiber diets, heavy smoking or a lack of exercise [5]. Hence, the promotion of physical and psychological well-being through exercise is likely to be of benefit to patients with schizophrenia.

Exercise is known to be of psychosocial benefit to patients. Levin and Gimino [6] showed that aerobic exercise reduces depression, anxiety and obsessive-compulsive symptoms in hospitalized patients with schizophrenia, relative to non-exercise-based treatment regimes. Similar interventions have been shown to improve mood, anxiety, depression, self-esteem, energy, concentration, quality of life and social interactions in a range of psychopathological disorders [7, 8]. A 10-week exercise program implemented by Faulkner and Sparkes was found to reduce auditory hallucinations, raise self-esteem and improve sleep patterns as well as general behaviors in patients with schizophrenia [9]. More recent research has uncovered anatomical changes associated with aerobic exercise, most notably increases in hippocampal volume [10], that may improve short-term memory in participants with schizophrenia. In addition to symptom-related outcomes, a lack of physical activity was associated with poorer health-related quality of life indices [11].

### Multiple benefits of Tai-chi as a physical and mental exercise

Based on Eastern health philosophies which focus on the interrelatedness of body and mind, Tai-chi is a form of moderate aerobic exercise that places emphasis on mental well-being [12, 13]. The basic principles of Tai-chi center on physical relaxation, mental alertness, movement sequencing and coordination [14]. In targeting the mind and body, Tai-chi may be particularly beneficial in patients with mental illnesses: there is now an increasing body of empirical evidence demonstrating its physical and mental benefits in clinical populations suffering from depression, posttraumatic stress disorders and traumatic brain injury [15–17]. The multiple benefits of Tai-chi were established in a recent review of 42 RCTs on Tai-chi [12]. Tai-chi was found to improve cardiovascular fitness, bone health, motor coordination, balance, flexibility, and to prevent falls [12, 18, 19]. Immunity, a crucial factor in physical health associated with illness prevention and prognosis, also improved after Tai-chi [20]. Furthermore, it was shown to facilitate psychological focus and relaxation [21], and to alleviate mood disturbances, anxiety, stress, tension, depression, anger and fatigue [16].

In addition to alleviating symptoms, Tai-chi can be used to target motor and cognitive deficits [22], which are subtle manifestations of cerebellar abnormalities and changes in neurological pathways [23]. Psychotropic drugs often induce severe motor deficits, including parkinsonism, dyskinesia and akathisia. Problems in movement and memory can potentially be alleviated by the practice of Tai-chi. The strength of the Wu-style (Cheng form) of Tai-chi [24] is its emphasis on rhythm and coordination. Participants name each movement while practicing; this process demands attention, concentration, memory and physical exertion within the exercise routine. Continual practice is highly encouraged after the completion of classes.

Despite efforts to investigate the impact of Tai-chi on psychological health outcomes, only one study has been published on the effectiveness of Tai-chi in schizophrenia. This study, conducted over 12 weeks, reported a reduction in negative symptoms in patients who had practiced Tai-chi [25]. It is postulated that the focus required for Tai-Chi encourages identification with the inner self, allowing problems of dissociation and attention, characteristic of schizophrenia, to be addressed. Nonetheless, little is known about the specific cognitive benefits of Tai-chi, or of the possible physiological mechanisms involved.

### Schizophrenic Symptoms and the HPA Axis

Salivary cortisol, a neuroendocrine indicator of stress and immunity, can provide insight into the possible physiological mechanisms underlying schizophrenia [16]. Patients with schizophrenia tend to have altered cortisol levels and stress responses, though studies conducted thus far have demonstrated both hyper- and hypo-function of the hypothalamic–pituitary–adrenal (HPA) axis which regulates cortisol levels [26]. Abnormally blunted cortisol responses have also been recorded in patients with schizophrenia [27]. Both hyper- and hypo-activity of the HPA axis have adverse effects on physical and psychological health. For patients with schizophrenia, increased cortisol levels are associated with more severe negative symptoms [28] and worse cognitive functioning in terms of verbal memory [29]; blunted cortisol responses are also associated with poorer quality of life [30]. While a previous study has demonstrated the effectiveness of Tai-chi in lowering salivary cortisol concentrations [31], the effects of lowered cortisol concentrations on symptoms, and other physical and psychological deficits that impair patient well-being, are virtually unknown. Alleviation of symptoms, other deficiencies and psychological health challenges can benefit patients’ overall functioning and quality of life. Tai-chi has been shown to confer these benefits in a number of chronic conditions [32].

### Research objectives

The present study protocol aims to explore the effects of Tai-chi on the symptoms (both positive and negative), motor and cognitive deficits (in terms of motor coordination, motor sequencing and memory), general functional disabilities, stress and salivary cortisol levels in patients with schizophrenia. The effectiveness of Tai-chi practice will be compared to an alternative exercise regimen and a waitlist control group.

## Methods/design

A non-blind, 3-arm randomized controlled trial (RCT) with waitlist control design will be used. Eligible participants will be randomized into (i) Tai-chi, (ii) exercise or (iii) waitlist control groups, on a 1:1:1 basis. The research is conducted in compliance with the Helsinki Declaration, and ethical approval has been obtained from Institutional Review Board of the University of Hong Kong/ Hospital Authority Hong Kong West Cluster (Ref: UW 11–481) before participant recruitment and randomization take place. Human Research Ethics Committee for Non-Clinical Faculties of the University of Hong Kong.

The exercise control will serve as a comparison to delineate the unique benefits of Tai-chi that cannot be accounted for by similar levels of moderate aerobic exercise. This comparison will facilitate a deeper understanding of the positive benefits of Tai-chi, such as attainment of mental tranquility and relaxation, which cannot be explained by physical exercise alone. While exercise is known to confer stress-reducing benefits, there are few studies that compare Tai-chi with alternative exercise interventions [33].

The waitlist control group will continue to receive regular medication and care during the intervention phase. Participants in this group will be offered the Tai-chi or exercise classes on a voluntary basis following completion of the study. Three assessment phases will be carried out for all groups at three time points: (i) baseline, (ii) 12th week (post-intervention time-point), and (iii) 24th week (maintenance time-point).

**Figure 1:**
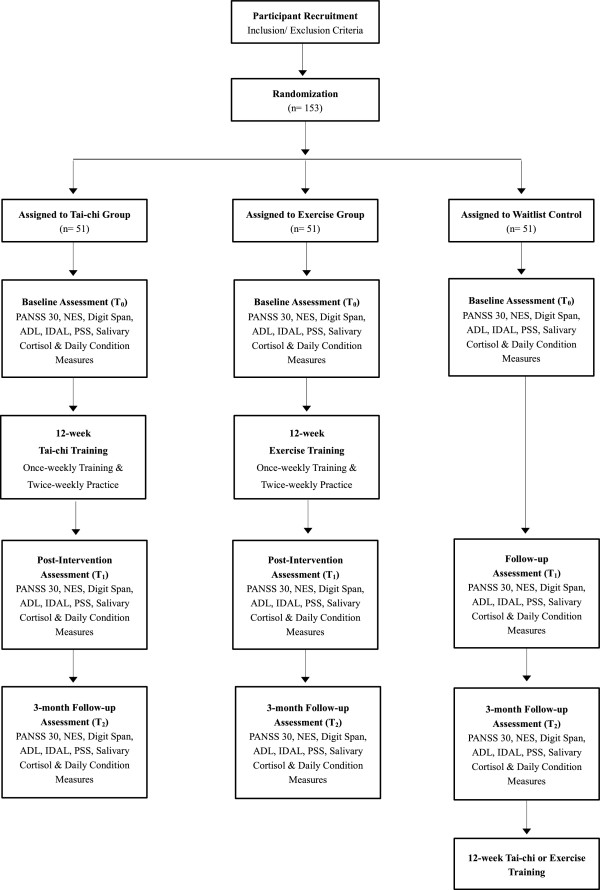
**Flow diagram of intervention/waitlist control and data collection points.**

Figure 1 illustrates the treatment received by the intervention group, the waitlist control condition received by the control group, and the data collection points of the study.

### Participant recruitment

Patients diagnosed with chronic schizophrenia, residing in a Hong Kong mental health rehabilitation complex providing both long-term care and halfway house services will be recruited to the study. Patients will be invited to participate by their respective social workers, based on specific inclusion and exclusion criteria.

Inclusion criteria will be as follows: a) fulfillment of DSM-IV TR criteria for schizophrenia, or diagnosis by a psychiatrist, b) age between 18 and 65 years, c) ability to understand and speak Cantonese, and d) no formal training in or regular practice of Tai-chi.

Exclusion criteria will be as follows: a) diagnosis of acute schizophrenia requiring hospitalization, b) presence of unstable or severe schizophrenic symptoms (e.g. persistent withdrawal) that would limit ability to interact or participate in the class, c) history of brain trauma or organic mental disorders (e.g. mental retardation or dementia), d) presence of physical disabilities, e) presence of other severe illnesses which may impair cognitive or visuo-motor function, cause physical pain or limit life expectancy to 10 years or less.

In determining the sample size, we have taken into consideration multiple independent variables, using multiple regression modeling with a medium effect size (f^2^) of 0.15, at 0.8 power and a significance level of 0.05. Experimental, clinical and demographic variables anticipated to affect the intervention outcome were included in the model. An attrition rate of approximately 25% is expected, based on prior community trials of Tai-chi interventions in elderly patients, and exercise programs for patients with severe mental illnesses [33]. Thus, a total of 153 participants will be targeted for the study.

Upon application of inclusion and exclusion criteria, a trained research assistant will obtain informed consent from participants at the rehabilitation complex. Eligible and consenting participants will then be randomized into one of the three treatment conditions, and baseline data collection will be arranged at least one week prior to the commencement of group programs.

### Intervention

The Tai-chi intervention is based on the first segment of the Wu-style Cheng-form Tai-chi chuan, comprising 22 simple movements [24]. The basic principal of this form is the emphasis on attention and coordination. Learning the names of each move will promote concentration and focus during practice. Results from the pilot study were used to optimize this program, to maximize learning and memory of the movements while encouraging practice. This will involve the use of flash cards with descriptive diagrams of the movement forms. The intervention will be conducted by mental health professionals with formal training in Tai-chi, who have attended 12 training sessions for instructors at the professional Tsui Woon Kwong Tai-chi Institute.

To design a control exercise regime of comparable intensity, a pilot control study was conducted with four schizophrenic patients (two male and two female) with former Tai-chi experience. The patients’ heart rates were measured by a portable heart rate monitor while they practiced a full set of Tai-chi movements. Based on the data collected, a qualified fitness instructor then devised a moderate aerobic exercise routine designed to achieve 50–60% maximal oxygen consumption (VO_2 max_) for the exercise group. The 1-hour exercise intervention, to be led by mental health professionals, will include a warm up, stretching and joint movements (15 minutes), walking (10 minutes), stepping (10 minutes), mild weight training (10 minutes) and cool down stretching (15 minutes).

For both the Tai-chi and exercise groups, a 1-hour weekly class will be held for 12 consecutive weeks. Sessions will be conducted in groups of 20. Participants will also be invited to a 45-minute, twice-weekly training session under the guidance of mental health professionals in between trainer-led classes. The waitlist group will receive routine care and be offered a similar Tai-chi or exercise class after the 24-week assessment period.

### Setting

All assessment and interventions will be conducted in a residential long-term care hostel for psychiatric patients in Hong Kong.

### Instruments

The PANSS is a psychiatric rating system that will be used to assess the positive and negative symptoms of schizophrenia exhibited in the week prior to assessment. It is based on information from the reports of family members or primary care staff, as well as a 40-minute semi-structured psychiatric interview with the patient. The patient will then be rated on a scale of 1 to 7 on 30 different symptoms pertaining to 3 different subscales—positive, negative, and general psychopathology—with internal consistency rats ranging from 0.73 to 0.83.

Assessments will be conducted by trained researchers using the following devices:

1. *Psychiatric Symptoms - The Positive and Negative Syndrome Scale (PANSS 30)* [34]
2. *Motor Coordination and Sequencing – Neurological Evaluation Scale (NES)* [35]
  2.1 Motor Coordination Subscale: motor coordination will be measured using four tasks, including (i) tandem walk, (ii) rapid alternation movements, (iii) finger/thumb opposition, and the finger-to-nose test [36]. The ability of the patient to perform each of the above tasks will be rated on a 3-point scale based on the number of mistakes.
  2.2 Sequencing of Complex Motor Acts Subscale: this will consist of four tasks associated with the sequencing of motor acts, including (i) the fist-ring test (right and left hands), (ii) the fist-edge-palm test (right and left hands), (iii) the Ozeretski test, and (iv) the rhythm tapping test [36]. Performance will be rated on a 3-point scale based on the number of mistakes.

3. *Digit Span – Wechsler Adult Intelligence Scale, Third Edition – Chinese version (WAIS-III)* [37] *–* The digit span sub-scale is composed of two parts: forward movement of digits, and backward movement of digits. Participants will be invited to repeat movements of 3*–*9 digits forward and 2*–*9 digits backward. Clinically, the sub-scale evaluates participants’ short-term memory, attention, and concentration.
4. *Barthel’s Activities of Daily Living (ADL) index – Chinese version (Barthel’s ADL)* [38] Barthel’s ADL index evaluates basic self-care. The index comprises 10 tasks, including feeding, transferring, grooming, toilet use, bathing, walking, climbing stairs, dressing, and bowel and bladder continence. The items are weighted according to a scheme developed by the authors.
5. *Lawton’s Instrumental Activities of Daily Living (IADL) scale – Chinese version* [39] – This self-report scale covers 8 domains of complex daily life, such as ability to use the telephone, shopping, food preparation, housekeeping, laundry, traveling on public transport, self-medication, and the ability to handle finances.
6. *Stress*
  6.1 Perceived stress scale (PSS) [40] – The PSS measures how often participants experience general stressfulness within a given month on a 10-item, 5-point Likert scale. Higher scores indicate greater stress. The Chinese version of the PSS was translated and utilized by the research team with good internal consistency.
  6.2 Salivary cortisol – Collection will be conducted by participants themselves under the guidance of mental health professionals. Saliva samples will be collected at four prescribed times (upon awakening, 30 minutes post-awakening, at 11:30 am and at 7:30 pm) using the collection device “Salivette” (Starstedt, Ag & Co., Nümbrecht, Germany), which includes a cotton swab to place under the tongue. Smoking, eating and drinking will be prohibited for half hour prior to saliva collection. The samples collected will be stored at −20°C until analysis.
  6.3 Daily condition measures with salivary cortisol – Measures of participants’ health-related behaviors and activities on the day of saliva collection will be collected in conjunction with salivary cortisol. These measures include (i) self-reported sleep quantity that day (hours of sleep at night and nap hours), (ii) subjective sleep quality rated on a scale of 1 to 10, (iii) smoking habit and the number of cigarettes consumed that day, (iv) alcohol/coffee drinking habits and the approximate amount consumed that day, and (v) subjective evaluation of dietary habits and the quality of diet on the day of collection rated on a scale of 1 to 10. All the above measures will affect the diurnal cortisol rhythm and hence must be taken into account during data interpretation.

7. *Socio-demographic and clinical informat*ion – Patients’ socio-demographic and clinical information will be obtained from personal and medical records. Socio-demographic data include age, gender, education level and marital status. The frequency and duration of exercise or relaxation practices will also be recorded. Clinical information includes the time since psychiatric diagnosis and condition severity, including acute and chronic extrapyramidal symptoms, particularly drug-related movement disorders such as parkinsonism, akathisia and dyskinesia [41]. Medication and other adjunctive treatments, including institutional care, will also be recorded. These factors can represent important confounding variables when modeling outcomes.

### Data analysis

#### Effectiveness of the Tai-Chi intervention

Repeated measures analysis of variance using SPSS software will be used to explore the effectiveness of the Tai-chi intervention over the three assessment time points, relative to the exercise and waitlist control groups. Ordinary least squares (OLS) regression will be used to assess the relationship between the dependent variables (outcomes) and independent variables (covariates). A model of the role of Tai-chi with respect to other influencing variables, such as clinical prognosis or frequency of practice, will deepen understanding of the true effects of Tai-chi in this clinical population. All levels of significance will be set at p < 0.05. Intention-to-treat analysis will be used to prevent participant selection bias due to study discontinuation.

#### Analysis of salivary cortisol

To obtain cortisol levels the saliva samples will first be thawed and centrifuged at 3000 rpm for 15 minutes at room temperature. The levels will be determined in the HKU Clinical Oncology Lab using an enzyme-linked immunosorbent assay (EIA) kit (Salimetrics Inc., USA). The assay sensitivity for this kit is 0.007 g/dl (i.e. 0.193 nmol/l) and the intra- and inter-assay coefficients of variation are 3% and 10%, respectively.

Due to the skewed distribution of salivary cortisol data, a natural logarithm will be used to normalize raw cortisol data. Mean cortisol levels will be calculated across four collection time points. Total cortisol levels will be expressed as area under the curve (AUC), and diurnal cortisol rhythm will be calculated by linear regression analysis of log-transformed cortisol levels at time of collection.

#### Relationship between cortisol levels and physical and psychological deficits

Using baseline data from all participants, correlation analyses will be used to explore the interrelationship between diurnal cortisol patterns and subjective stress, psychiatric symptoms, motor deficits, memory test and general functioning.

To trace individual changes in salivary cortisol levels over time, and to evaluate the complex relationships between different variables, the two-level individual growth curve model [42] and MPlus software will be used for analysis. This method is a variant of multiple regression modeling appropriate for the nested structure of our data sets. In the study, salivary cortisol measures at four time points over the day are nested within participants.

## Discussion

While physical exercise has been researched extensively, this RCT will represent one of the first investigations into mind-body exercises in patients with schizophrenia. The results of a pilot study demonstrated attenuated deterioration in motor coordination and daily functioning in patients practicing Tai-chi. With a larger sample size, and using an exercise group for comparison, the aims of this RCT are to confirm previous findings and to provide new insight into how Tai-chi may provide unique benefits to patients with schizophrenia, above the effects of moderate aerobic exercise. Analysis of salivary cortisol levels will elucidate the effect of Tai-chi on physiological stress. The results will establish the feasibility and efficacy of integrating mind-body exercises into routine care as complementary treatment for psychiatric conditions.
